# Clinical outcomes in patients admitted to hospital with cervical spine fractures or with hip fractures

**DOI:** 10.1007/s11739-020-02567-x

**Published:** 2020-11-26

**Authors:** Joshua Baxter, Radcliffe Lisk, Ahmad Osmani, Keefai Yeong, Jonathan Robin, David Fluck, Christopher Henry Fry, Thang Sieu Han

**Affiliations:** 1 Birmingham Medical School, College of Medical and Dental Sciences, University of Birmingham, Birmingham, B15 2TT UK; 2 Department of Orthogeriatrics, Ashford and St Peter’s NHS Foundation Trust, Guildford Road, Chertsey, KT16 0PZ Surrey UK; 3 Department of Medicine, Ashford and St Peter’s NHS Foundation Trust, Guildford Road, Chertsey, KT16 0PZ Surrey UK; 4 Department of Cardiology, Ashford and St Peter’s NHS Foundation Trust, Guildford Road, Chertsey, KT16 0PZ Surrey UK; 5 School of Physiology, Pharmacology and Neuroscience, University of Bristol, Bristol, BS8 1TD UK; 6 Institute of Cardiovascular Research, Royal Holloway, University of London, Egham, TW20 0EX Surrey UK

**Keywords:** Geriatrics, Pressure ulcers, Length of stay, Mortality, Discharge destination

## Abstract

Patients admitted with a cervical fracture are twice as likely to die within 30 days of injury than those with a hip fracture. However, guidelines for the management of cervical fractures are less available than for hip fractures. We hypothesise that outcomes may differ between these types of fractures. We analysed 1359 patients (406 men, 953 women) with mean age of 83.8 years (standard deviation = 8.7) admitted to a National Health Service hospital in 2013–2019 with a cervical (7.5%) or hip fracture (92.5%) of similar age. The association of cervical fracture (hip fracture as reference), hospital length of stay (LOS), co-morbidities, age and sex with outcomes (acute delirium, new pressure ulcer, and discharge to residential/nursing care) was assessed by stepwise multivariate logistic regression. *Acute delirium without history of dementia* was increased with cervical fractures: odds ratio (OR) = 2.4, 95% confidence interval (CI) = 1.3–4.7, age ≥ 80 years: OR = 3.5 (95% CI = 1.9–6.4), history of stroke: OR = 1.8 (95% CI = 1.0–3.1) and ischaemic heart disease: OR = 1.9 (95% CI = 1.1–3.6); *pressure ulcers* was increased with cervical fractures: OR = 10.9 (95% CI = 5.3–22.7), LOS of 2–3 weeks: OR = 3.0 (95% CI = 1.2–7.5) and LOS of ≥ 3 weeks: OR = 4.9, 95% CI = 2.2–11.0; and *discharge to residential/nursing care* was increased with cervical fractures: OR = 3.2 (95% CI = 1.4–7.0), LOS of ≥ 3 weeks: OR = 4.4 (95% CI = 2.5–7.6), dementia: OR = 2.7 (95% CI = 1.6–4.7), Parkinson’s disease: OR = 3.4 (95% CI = 1.3–8.8), and age ≥ 80 years: OR = 2.7 (95% CI = 1.3–5.6). In conclusion, compared with hip fracture, cervical fracture is more likely to associate with acute delirium and pressure ulcers, and for discharge to residency of high level of care, independent of established risk factors.

## Introduction

Hip fracture is a disabling condition affecting many older individuals worldwide, with incidence rates being greatest among high income countries, particularly those further from the equator [1]. The estimated lifetime risk of a hip fracture is 23% in European women and 11% in European men [2]. In 1990, the global number of hip fractures was 1.26 million. Because of the steep rise in the rates of hip fractures with age and the growing ageing population, their number is expected to rise to 4.5 million by 2050 [3]. Hip fracture is a prognostic indicator of mortality and disability. A substantial proportion of survivors require long-term high levels of care, imposing an enormous burden on health-care systems [1, 3–5]. The mean cost for an index hospitalisation has been estimated to be over US $10,000, and health and social care costs almost $44,000 at 1 year [6].

By contrast, cervical spine fractures occur far less frequently than hip fractures [7], but the incidence also increases with age [8] and has been rising in recent years. A study of 167,278 older US adults admitted with cervical fractures showed a big increase in the rates of hospitalisation (from 26/100,000 to 68/100,000) and mortality (from 3/100,000 to 6/100,000) between 2001 and 2010 [9]. Mortality among hospital patients with cervical fractures has been reported to be 8–14% [9, 10], and 28–37% within 1 year of the fracture [11–14]. Unlike hip fractures, the majority of cervical fractures are treated non-surgically. However, supportive treatment such as cervical spine immobilisation appears to associate with a number of complications including pressure sores, raised intracranial pressure, swallowing and breathing difficulties, and exacerbation of delirium [15].

A study of over a million patients showed that patients who sustained a cervical fracture were twice as likely to die within 30 days of the injury than those who sustained a hip fracture [14]. However, guidelines for the management of cervical fractures is less comprehensive compared to those for hip fractures [7, 16]. Because the management approaches to these two age-related acute conditions remain highly contrasting [14], we hypothesise that post-fracture outcomes in patients with cervical fractures may differ from those in patients with hip fractures.

## Methods

### Study design, participants and setting

We conducted a cross-sectional study of older individuals (aged 60–103 years) admitted with cervical and hip fractures to a National Health Service hospital between 2013 and 2019, serving a catchment population of over 410,000 people.

### Data collection

Data were prospectively collected by a Trauma Coordinator for patients admitted with a hip or cervical fracture from the time of admission to discharge [17–19]. Co-morbidities were identified from electronic record databases by disease codes defined by the International Classification of Diseases 10 [20]. Data consisted of clinical characteristics and care quality was updated regularly into a database managed by the lead orthogeriatrician to ensure completeness and accuracy of data entry. Demographic and clinical information included age, sex, residency prior to admission, dates of admission and discharge from which length of stay (LOS) in hospital was calculated, mental status (acute delirium) at admission, new pressure ulcers developed during admission and discharge destination.

### Categorisation of variables

Pressure ulcers were defined as grade 2 or above and newly acquired in hospital. LOS in hospital was categorised into three groups: < 2 weeks, 2–3 weeks and ≥ 3 weeks, and age into two groups: < 80 and ≥ 80 years. Change to discharge destination was defined as those who came from their own home before hospital admission, but were transferred to places of higher level of care including rehabilitation units, residential or nursing care. Acute delirium was based on standard clinical assessment tool and only patients without a history of dementia were included for this particular analysis.

### Statistical analysis

Continuous group data are summarised as mean values ± standard deviation (SD). Differences between categorical outcome variables were assessed by Chi-squared tests. Stepwise multivariate logistic regression was used to assess the association of: the types of fractures; LOS; chronic co-morbidities including dementia, stroke, Parkinson’s disease, ischaemic heart disease and diabetes; age and sex (dependent variables) with outcomes including: acute delirium; new pressure ulcers; and discharge to residence of high level of care (residential or nursing care). All independent variables were entered simultaneously; only variables associate significantly with outcome measures are automatically selected by this stepwise regression technique and presented in our results. Analyses were performed using IBM SPSS Statistics, v25.0 (IBM Corp., Armonk, NY).

## Results

The data of a total of 1359 patients (406 men, 953 women) aged 83.8 years (SD ± 8.7) were analysed. Most patients came from their own home (81.3%), followed by residential/nursing care (12.0%) and rehabilitation (6.7%). 102 (7.5%) patients were admitted with cervical fractures with mean age of 82.7 years (SD ± 9.8) and 1257 (350 men, 907 women) patients admitted with hip fractures with mean age of 83.9 years (SD ± 8.6). There was no age difference between the two groups of fractures (*p* = 0.214). Falls were the main cause of fractures, occurring in 95.5% of hip fractures and 93.1% of cervical fractures. The remaining causes of cervical fractures (6.9%) were patients involved in road traffic or other high-impact accidents. There were 28.6% of patients with co-exiting dementia, 15.2% with stroke, 4.2% with Parkinson’s disease, 9.9% with ischaemic heart disease and 13.5% with diabetes. The proportions of patients with hospital LOS < 2 weeks, 2–3 weeks and ≥ 3 weeks were 68.7, 15.7 and 15.6%, respectively. There were 2.6% of patients who acquired a new ulcer in hospital. Among those who came from their own home, 53.8% were discharged back home, 28.2% to rehabilitation and 18.0% to residential/nursing care (Table 1).

**Table 1:** Subject characteristics of 102 (56 men, 46 women) patients admitted with cervical fractures with mean age of 82.7 years (SD ± 9.8) and 1257 (350 men, 907 women) patients admitted with hip fractures with mean age of 83.9 (SD ± 8.6)

	%
Demographics	
Admitted from home: nursing/residential care: rehabilitation	81.3: 12.0: 6.7
Men: women	29.9: 70.1
Age < 80 years: ≥ 80 years	28.6: 71.4
Outcomes	
Cervical fractures: hip fractures	7.5: 92.5
Acute delirium without dementia	10.6
LOS in hospital < 2 weeks: 2–3 weeks: ≥ 3 weeks	68.7: 15.7: 15.6
Pressure ulcers	2.6
Discharged to own home: rehabilitation: nursing/residential care	53.8: 28.2:18.0
Coexisting morbidities	
Dementia	28.6
Stroke	15.2
Parkinson’s disease	4.2
Ischaemic heart disease	9.9
Diabetes	13.5

A total of 1359 patients (406 men, 953 women) aged 83.8 years (SD ± 8.7)

**Fig. 1:**
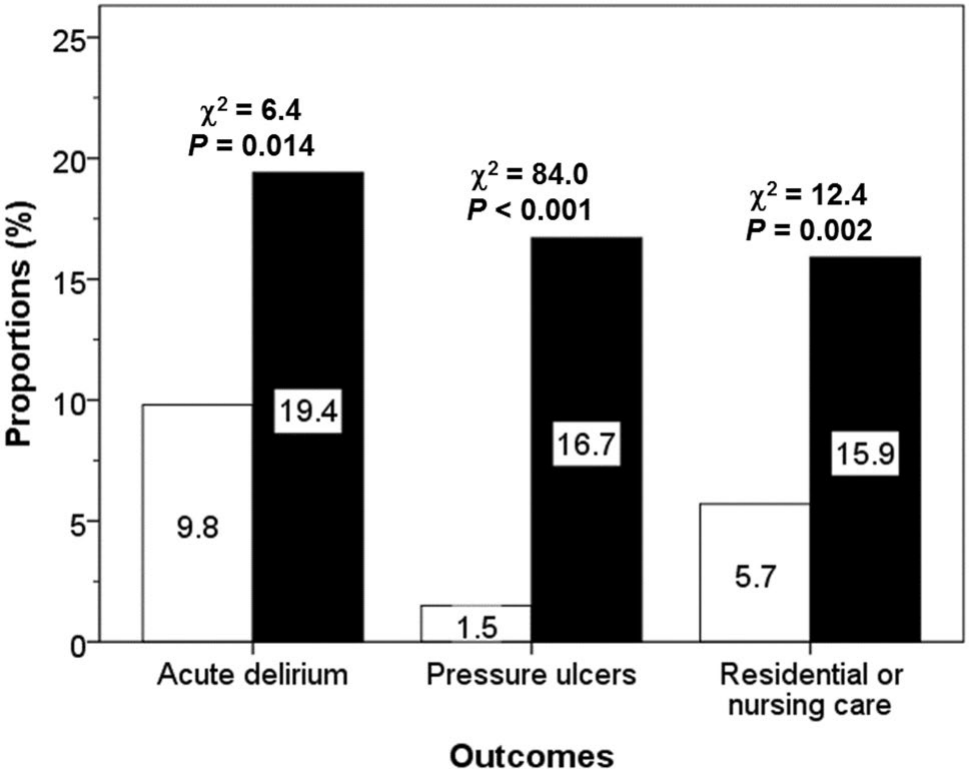
Rates of acute delirium on admission, new pressure ulcers and discharge to residential/nursing care in patients admitted with cervical (open bars) or with hip fractures (black bars): Chi-squared tests showing group differences for each outcome

Compared with patients admitted with hip fractures, patients admitted with cervical fractures had higher rates of acute delirium without a history of dementia on admission (9.8% versus 19.4%, *p* = 0.014), development of a new pressure ulcer in hospital (1.5% versus 16.7%, *p* < 0.001), and discharge to residential or nursing care (5.7% versus 15.9%, *p* = 0.002) (Fig. 1).

The rates of acute delirium without a history of dementia were also higher in patients with a history of stroke, ischaemic heart disease, and aged ≥ 80 years. The rates of new pressure ulcers acquired in hospital were higher in patients with cervical fractures than those with hip fractures, staying in hospital ≥ 2 weeks, and those with a history of diabetes. Among those who were admitted from their own home, higher proportions of those who were discharged to residential/nursing care were observed for patients admitted with cervical fractures than those with hip fractures, staying in hospital ≥ 2 weeks, and patients with underlying dementia, Parkinson’s disease or diabetes, and those aged ≥ 80 years (Table 2).

**Table 2:** Proportions of patients with different outcomes

	Acute delirium (%)*	*χ*^2^	*p*	Pressure ulcers (%)	*χ*^2^	*p*	Discharge to residential or nursing care (%)	*χ*^2^	*p*
Length of stay < 2 weeks	8.4	18.5	< 0.001	1.3	22.9	< 0.001	3.2	60.8	< 0.001
Length of stay 2–3 weeks	12.6			4.7			8.1		
Length of stay ≥ 3 weeks	23.1			6.6			20.6		
No dementia	–	–	–	2.8	0.2	0.391	4.3	30.9	< 0.001
Dementia	–			2.3			14.9		
No stroke	9.5	7.1	0.009	2.2	3.4	0.061	6.1	0.5	0.299
Stroke	17.6			4.4			7.6		
No Parkinson’s disease	10.4	1.4	0.176	2.5	0.2	0.438	5.9	8.7	0.011
Parkinson’s disease	16.7			3.5			17.5		
No ischaemic heart disease	9.8	5.6	0.019	2.6	0.1	0.488	6.1	0.7	0.266
Ischaemic heart disease	18.0			3.0			8.2		
No diabetes	11.0	0.6	0.282	2.2	6.5	0.016	5.8	3.8	0.046
Diabetes	8.6			5.5			10.1		
Age < 80 years	4.5	17.3	< 0.001	1.5	2.6	0.074	2.6	12.2	< 0.001
Age ≥ 80 years	13.8			3.1			8.2		
Women	10.2	0.5	0.280	2.5	0.2	0.384	6.7	0.6	0.261
Men	11.7			3.0			5.4		

*Only patients without dementia were included in delirium analysis

Four patients (3.9%) admitted with cervical fractures developed pressure ulcers in the neck area

Stepwise multivariate logistic regression showed that *acute delirium without history of dementia* was increased with cervical fractures: odds ratio (OR) = 2.4, 95% confidence interval (CI) = 1.3–4.7, aged ≥ 80 years: OR = 3.5 (95% CI = 1.9–6.4), history of stroke: OR = 1.8 (95% CI = 1.0–3.1) and ischaemic heart disease: OR = 1.9 (95% CI = 1.1–3.6); and *pressure ulcers* was increased with cervical fractures: OR = 10.9 (95% CI = 5.3–22.7), LOS between 2 and 3 weeks: OR = 3.0 (95% CI = 1.2–7.5) and LOS ≥ 3 weeks: OR = 4.9, 95% CI = 2.2–11.0. After excluding four cases (3.9%) with collar-related ulcers in patients with cervical fractures, pressure ulcers in cervical fractures remained greater than in hip fractures: OR = 9.4 (95% CI = 4.9–21.3). Among those who came from their own home, *discharge to residential/nursing care* was increased with cervical fractures: OR = 3.2 (95% CI = 1.4–7.0), LOS ≥ 3 weeks: OR = 4.4 (95% CI = 2.5–7.6), dementia: OR = 2.7 (95% CI = 1.6–4.7), Parkinson’s disease: OR = 3.4 (95% CI = 1.3–8.8), and aged ≥ 80 years: OR = 2.7 (95% CI = 1.3–5.6) (Table 3). The use of age as continuous variable did not substantially change the ORs for all outcomes above.

**Table 3:** Predictive models constructed by multivariate stepwise logistic regression simultaneously analysing cervical and hip fractures with all established risk factors (shown in Table 2) to predict clinical outcome measures

Acute delirium without a history of dementia			
Risk factors	OR	95% CI	*p*
Hip fractures	1	–	–
Cervical fractures	2.4	1.3–4.7	0.008
Age < 80 years	1	–	–
Age 80 ≥ years	3.5	1.9–6.4	< 0.001
No stroke	1	–	–
Stroke	1.8	1.0–3.1	0.041
No ischaemic heart disease	1	–	–
Ischaemic heart disease	1.9	1.1–3.6	0.033

Pressure ulcers			
Risk factors	OR	95% CI	*p*
Hip fractures	1	–	–
Cervical fractures*	10.9	5.3–22.7	< 0.001
Length of stay < 2 weeks	1	–	–
Length of stay 2–3 weeks	3.0	1.2–7.5	0.022
Length of stay ≥ 3 weeks	4.9	2.2–11.0	< 0.001

Discharged to residential/nursing care			
Hip fractures	1	–	–
Cervical fractures	3.2	1.4–7.0	0.004
Length of stay < 2 weeks	1	–	–
Length of stay ≥ 3 weeks	4.4	2.5–7.6	< 0.001
No dementia	1	–	–
Dementia	2.7	1.6–4.7	< 0.001
No Parkinson’s disease	1	–	–
Parkinson’s disease	3.4	1.3–8.8	0.012
Age < 80 years	1	–	–
Age 80 ≥ years	2.7	1.3–5.6	0.010

*Excluding four cases with collar-related ulcers: OR = 9.4 (95% CI = 4.9–21.3)

## Discussion

The present study observed that compared with hip fracture, cervical fracture was more likely to associate with acute delirium by 2.4-fold, pressure ulcers by 10.9-fold and discharge to residential/nursing care by 3.2-fold, independent of other established risk factors. Our findings are novel since, as far as we are aware, no previous studies have simultaneously analysed the relative contributions of the types of fracture towards clinical outcomes. More research is warranted to identify underlying causes of these poor outcomes to establish and implement guidelines in clinical practice which may further improve the management and minimise the level of risk in such group of patients.

The observation of more patients admitted with hip fractures (92.5%) than those with cervical fracture (7.5%) over the same period of study are almost the same as figures (93.8% versus 6.2%) reported from a large study of US patients [14]. The proportions of women and men (72.2% versus 27.8%) admitted with hip fractures were similar to figures reported from studies of high income countries [4] and the slightly greater number of men than women (54.9% versus 45.1%) with cervical fractures is also similar to those reported from other studies [9, 12, 14, 21]. Most of the cervical fractures were due to falls, which are again consistent with previous reports [9]. On the other hand, the aetiologies of cervical fractures in younger adults (< 60 years) are very different, mostly arising from contact and collision sports such as rugby [22], football, gymnastics [23] and diving [24], as well as road traffic accidents [8].

Although cervical and hip fractures are both age-related acute conditions, approaches to their management are highly contrasting. In the UK, national audit and guidelines for management of hip fractures have been well established and updated regularly [16]. The emphasis on specialist orthogeriatric oversight within 72 h, early restorative surgery and mobilisation by physiotherapists 1 day after surgery reflect an intensive intervention strategy. The most recent NHFD report showed the national average for achieving key performance indicators ranged between 69 and 90%. The delivery of these interventions has coincided with progressive reduction in 30-day mortality since 2007 [16].

By contrast, the nationally reported performance and ongoing audit and review of cervical fracture management does not seem to be subject to any form of national organised review and there are no existing key performance indicators or other targets set out for this group of patients in the UK. Most existing literature appears to focus on immediate management such as neck immobilisation and rapid diagnosis of cervical fractures [7, 25]. Management of patients with cervical fractures is usually conservative, with bed rest and immobilisation forming the mainstay of therapy. Surgery for cervical fractures is much less commonly performed than for hip fractures: only in circumstances such as unstable fractures or spinal cord compression [26, 27]. A number of factors may influence the decision for not operating cervical fractures, including the greater complexity of the cervical spine and age of the patients. Compared with patients who did not receive surgery for cervical fractures, early mortality rates (at 1 month or at 3 months) after surgery were lower in the younger group (65–74 years), but higher in older individuals (over 85 years) [12]. Bed rest seems to be associated with a number of complications including pressure ulcers, thromboembolism and residual immobility. Although the standard practice is to advise the patient to sit in a chair for a certain period of time as soon as possible, this varies widely between National Health Service hospitals. There is therefore an urgent need for standardised national guidelines on management of cervical fractures similar to those for management of hip fractures.

Orthotic devices, such as C-collars, Miami J-collars and halo-fixation devices are commonly used in cervical fractures [28]. Collars are responsible for device-related pressure ulcers due to an increased load on the soft tissue of the neck and surround areas that is usually not adapted for bearing pressure [29]. The commonly observed delirium in patients with cervical fractures presents a major challenge in management of this group of patients as many are not able to tolerate support collars, and delirium may be exacerbated by their use. It should be recognised that the association of cervical fractures and co-morbidities with delirium should not be interpreted as there being causal links, as their relationships are often reciprocal.

The observation of higher proportions of patients with cervical fractures being transferred to residence of higher level of care than those of patients with hip fractures suggests poorer recovery among patients with cervical fractures. Previous studies have found that cervical fractures led to long term disability in over half of patients [26, 27]. The majority of patients with cervical fractures acquired pressure ulcers in other areas not caused by the collar, and the higher proportion of non-collar related pressure pressures in these patients than that of patients with hip fractures. It is possible that these differences may be due to immobility and severity, or current management approach of cervical fractures. Pressure ulcers tend to develop rapidly in patients who are unable to shift their weight distribution regularly, which may be the case with cervical fractures. Pressure ulcers are an important indicator of quality of patient care. They impact adversely on patient safety and experience and carry a financial burden for the National Health Service, with the annual cost of £1382 for wound care per patient with a category 1 pressure ulcer, rising to an excess of £8500 per patient with a category ≥ 2 pressure ulcer [30].

There are certain limitations in our study including its cross-sectional design and relatively small numbers of patients with cervical fractures. Thus other outcomes such mortality could not be examined and this caveat should be appreciated when interpreting our findings. Future studies with larger numbers of patients with cervical fractures would be helpful to support our findings with greater confidence. The strengths of our study lie in its wide range of clinical measures for multivariate stepwise analysis to identify the most influential factors, including chronic conditions that indicate the individual’s frailty, and allow adjustment of potential confounding effects from each other. The demographic distributions such as age and sex, and proportions of different types of fractures were very similar to those reported for patients admitted to hospital in high income countries (see above), and thus provide confidence in the findings from our study.

In conclusion, compared with hip fracture, cervical fracture is more likely to associate with acute delirium and pressure ulcers, and for discharge to residency of high level of care, independent of established risk factors.

## Abbreviations

CI: Confidence interval
LOS: Length of stay
OR: Odds ratio
SD: Standard deviation
